# Probabilistic Updating of Structural Models for Damage Assessment Using Approximate Bayesian Computation

**DOI:** 10.3390/s20113197

**Published:** 2020-06-04

**Authors:** Zhouquan Feng, Yang Lin, Wenzan Wang, Xugang Hua, Zhengqing Chen

**Affiliations:** 1 Key Laboratory of Wind and Bridge Engineering of Hunan Province, College of Civil Engineering, Hunan University, Changsha 410082, China; linyang@hnu.edu.cn (Y.L.); wenzanwang@hnu.edu.cn (W.W.); cexghua@hnu.edu.cn (X.H.); zqchen@hnu.edu.cn (Z.C.); 2 State Key Laboratory of Advanced Design and Manufacturing for Vehicle Body, Hunan University, Changsha 410082, China

**Keywords:** model updating, damage detection, modal parameter, approximate Bayesian computation, subset simulation

## Abstract

A novel probabilistic approach for model updating based on approximate Bayesian computation with subset simulation (ABC-SubSim) is proposed for damage assessment of structures using modal data. The ABC-SubSim is a likelihood-free Bayesian approach in which the explicit expression of likelihood function is avoided and the posterior samples of model parameters are obtained using the technique of subset simulation. The novel contributions of this paper are on three fronts: one is the introduction of some new stopping criteria to find an appropriate tolerance level for the metric used in the ABC-SubSim; the second one is the employment of a hybrid optimization scheme to find finer optimal values for the model parameters; and the last one is the adoption of an iterative approach to determine the optimal weighting factors related to the residuals of modal frequency and mode shape in the metric. The effectiveness of this approach is demonstrated using three illustrative examples.

## 1. Introduction

Structural health monitoring (SHM) refers to the damage detection and characterization of engineering structures, which is very important for guaranteeing the safety and serviceability of structures. In recent years, this research field has gained much attention and has made many ground-breaking improvements. The SHM approaches can be roughly divided into two sorts: local approaches and global approaches. The former ones refer to the methods of assessing the highly localized behavior of structures (such as strain at a single location), which includes many local forms of non-destructive evaluation (NDE) [1,2,3]. The latter ones refer to the methods of assessing the global behavior of structures (such as modal parameters), which includes many vibration-based damage assessment methods [4]. A large portion of these damage assessment methods are built using model updating: based on the experimental (modal) data, structural damages are detected, located, and quantified by calibrating the stiffness parameters of structural model [5].

Structural model updating based on modal data is a process of correcting the initial model to a better model that reflects the dynamic behavior of the structure. Besides damage assessment, structural model updating has also been widely used in model validation, response prediction, structural control, etc. [6]. Due to a variety of uncertainties such as modeling error and measurement noise, the discrepancy between the theoretical finite element (FE) model and the actual structure is inevitable, so it is often necessary to update the initial/prior FE model. This topic has become a research hotspot in recent years [7,8,9,10,11,12,13,14,15]. The methods of model updating can be broadly divided into two types: deterministic methods and probabilistic methods. In deterministic methods, one’s objective is to find a single set of optimal model parameters by using an optimization technique to minimize a single-objective function [9,16] or multiple objective functions [17,18] that measure the goodness of fit between the quantities of model prediction and measurement, while the probabilistic approaches estimate the statistical distributions of the model parameters (not only the optimal parameters but also their uncertainties) given a family of possible models [19,20]. By providing well-defined formulations, the deterministic methods can effectively obtain a set of optimal values of model parameters for a well-posed identification or updating problem; nevertheless, they usually fail to deliver satisfactory results when many uncertainties are present. In contrast, probabilistic methods have more advantages in dealing with uncertainty because they can provide the probability distribution of model parameters, as well as the plausibility of the model itself based on measured data.

In recent years, probabilistic methods have been more and more popular in model updating, among which the Bayesian method is one of them. In this method, the available information involved in the measured data is utilized for statistical inference by constructing a suitable likelihood function [7,19]. In the updating framework of the Bayesian approach, the posterior distribution of model parameters is expressed as a product of the prior distribution and the likelihood function. The likelihood function represents the probability of obtaining measured data based on a given stochastic prediction model. Due to a lack of prior information, the prior distribution is often a non-informative distribution, so the likelihood function is crucial to the posterior distribution. If different probability models are assumed in the likelihood function, the subsequent posterior distribution will be different. Usually, the likelihood function is formulated as normal distribution based on the maximum entropy principle [19]. However, challenges still exist in construction of the likelihood function. For some complex models, the explicit expression of the likelihood function may be difficult or even impossible. Another difficulty is the correlation formula of the model parameters, i.e., it is difficult to explicitly express these correlations in the joint probability density function (PDF) of the likelihood function [21]. For finite-element model updating, the likelihood is a probabilistic model for the error between predictions and observations. Most often, a zero-mean uncorrelated Gaussian prediction error is assumed, but this assumption may be questionable in some cases. For model updating in the time domain, the prediction errors of time histories may be non-Gaussian, and they may also show temporal correlation in adjacent time instants; on the other hand, the prediction errors corresponding to different measurements may be correlated as well. For model updating in the frequency domain, the components of a mode shape may show spatial correlation when densely populated sensor grids are used. The assumption of prediction error correlation highly influences the results of Bayesian model updating, and it is a challenging task for us to choose a suitable correlation structure of prediction errors in the likelihood function.

In order to solve above problems, approximate Bayesian computation (ABC) was well-conceived to steer clear of the explicit expression of likelihood function, using a simulation-based approach instead [22]. Thus, it is a likelihood-free Bayesian method. ABC does not directly use the observed data to make statistical inference according to Bayes’ theorem, but it obtains the model prediction output through sampling and simulation, and the discrepancy between the predicted output and the observed data is acceptable under certain metric. Therefore, an approximate likelihood function is defined as the probability that the simulated predicted output falls into the neighboring region of the observed data vector. Obviously, the accuracy of this approximation depends on the selected tolerance level ϵ. In theory, when ϵ → 0, the algorithm gives the exact posterior distribution, but this cannot be done in practice. To get a good approximation of the posterior distribution in the ABC method, the tolerance parameter ϵ has to be small enough so that only the predicted output very close to the observed data vector is accepted. As a result, this becomes a simulation problem for rare events. If a Monte Carlo simulation is used, a large number of model prediction outputs must be calculated as candidate samples in order to obtain an acceptable sample size in the given approximate region of observed data. Jin and Jung presented a feasibility study on structural model calibration by using a Markov chain Monte Carlo (MCMC)-based ABC [23]. Fang et al. adopted ABC incorporated with the Metropolis–Hastings sampling (MHS) algorithm and stochastic response surface (SRS) for probabilistic damage identification [24]. Abdessalem et al. presented ABC sequential Monte Carlo (SMC) [25] and ABC ellipsoidal nested sampling (NS) [26] as efficient tools for model selection and parameter estimation in structural dynamics. Though MCMC can reduce computation cost, its efficiency is still not very high due to its slow chain initialization and convergence speed. In order to improve the efficiency of the ABC, Chiachio et al. [27] recently put forward the efficient subset simulation technique [28] into the ABC framework, naming it as ABC-SubSim. The basic idea is to take the nested descending sequence in the subset simulation as the approximate region that is increasing close to the observed data vector [29,30].

This paper proposes a new probabilistic structural model updating approach based on the ABC-SubSim for structural model updating using modal data. Obviously, the accuracy of the method depends on the selection of the tolerance level ϵ on the chosen metric. First, an appropriate metric should be chosen to measure the closeness of the predicted modal data vector and the observed modal data vector. The tolerance parameter ϵ should be sufficiently small to make a good approximation of the posterior distribution. However, the question of how small is sufficient enough is also questionable. To this end, we propose some new stopping criteria to cease the subset simulation, where the tolerance parameter ϵ for the metric is not predetermined. On the other hand, by using subset simulation, although the approximation region of the observed data vector for a given tolerance level can be easily reached, a refined optimal value may not be easily obtained due to the stochastic exploration nature of the subset simulation. To this end, we propose a hybrid optimization scheme that combines the subset simulation with a faster local optimization technique to obtain a more refined optimal value of the model parameter vector. The last contribution of this paper is that we adopt an iterative approach to determine the optimal weighting factors relating to the residuals of modal frequencies and mode shapes in the metric.

The remainder of the paper is organized as follows. The ABC-SubSim is reviewed in Section 2, where the choice of some important parameters such as intermediate tolerances, conditional probability and variance of proposal distribution is discussed. The procedures of model updating with ABC-SubSim is presented in Section 3. Section 4 presents a damage assessment approach using model updating. Three illustrative examples are presented in Section 5, and conclusions are drawn in Section 6.

## 2. Recap of ABC-SubSim

### 2.1. Approximate Bayesian Computation

Based on a set of measured data $y\in D\subset {\mathbb{R}}^{l}$, the parameter of interest $\theta \in \Theta \subset {\mathbb{R}}^{d}$ in a parameterized model class *M* can be updated from prior distribution to posterior distribution by Bayesian inference. On the basis of Bayes’ theorem, the posterior PDF p(θ|y,M) of the parameters *θ* in model *M* is written as follows [19]:

(1) p(θ|y,M)=cp(y|θ,M)p(θ|M)

where p(θ|y,M) is a posterior distribution of θ after taking measured data y into account, *c* is a normalizing constant to make the integral of posterior PDF to be one, p(y|θ,M) is the likelihood function which represents the distribution of the observed data y conditional on its parameters *θ*, and p(θ|M) is a prior distribution of the parameters *θ* before any data are observed. For a given model, we only care about the parameters in the model, not the selection of the model, so the conditioning on model class *M* can be removed in the notation. Although this Bayesian framework is powerful for uncertainties treatment, construction of the likelihood function is still a challenge in some cases. Approximate Bayesian computation methods circumvent the explicit expression of the likelihood function by using simulation-based methods. Let $x\in D\subset {\mathbb{R}}^{l}$ denote a dataset sampled from p(·|θ,M), the ABC algorithms take the approximate likelihood function $P_{\epsilon }\left(y|\theta \right)=\int P\left(x\in N_{\epsilon }\left(y\right)|x\right)p(x|\theta )dx$, where $N_{\epsilon }\left(y\right)$ is an approximate neighborhood of observed data for a chosen tolerance level ϵ on the given metric *ρ* and $N_{\epsilon }\left(y\right)=\left\{x\in D:\rho \left(\eta \left(x\right),\eta \left(y\right)\right)\leq \epsilon \right\}$. *η*(·) is a low-dimensional vector of summary statistics that provides a comparison of the closeness of *x* and *y*. From the Bayes’ theorem, the approximate posterior is given by

(2) $$p_{\epsilon }\left(\theta ,x|y\right)\propto P(x\in N_{\epsilon }\left(y\right)|x)p(x|\theta )p\left(\theta \right)$$

where $P(x\in N_{\epsilon }\left(y\right)|x)=I_{N_{\epsilon }\left(y\right)}\left(x\right)$ is an indicator function that assigns a value of one when ρ(η(x),η(y))≤ϵ and zero otherwise. The marginal approximate posterior:

(3) $$p_{\epsilon }\left(\theta |y\right)\propto P(x\in N_{\epsilon }\left(y\right)|\theta )p\left(\theta \right)$$

It should be noted that the quality of the approximated posterior in Equations (2) and (3) depends on an appropriate choice of the metric ρ, the tolerance level ϵ and the summary statistic *η* [31,32].

In order to obtain a good approximate posterior distribution, the tolerance ϵ need to be small enough, which leads to the simulation of rare events. Direct Monte Carlo simulation is inefficient, and although MCMC sampling can improve the efficiency, it is still not very efficient.

### 2.2. The ABC-SubSim Algorithm

**Figure 1:**
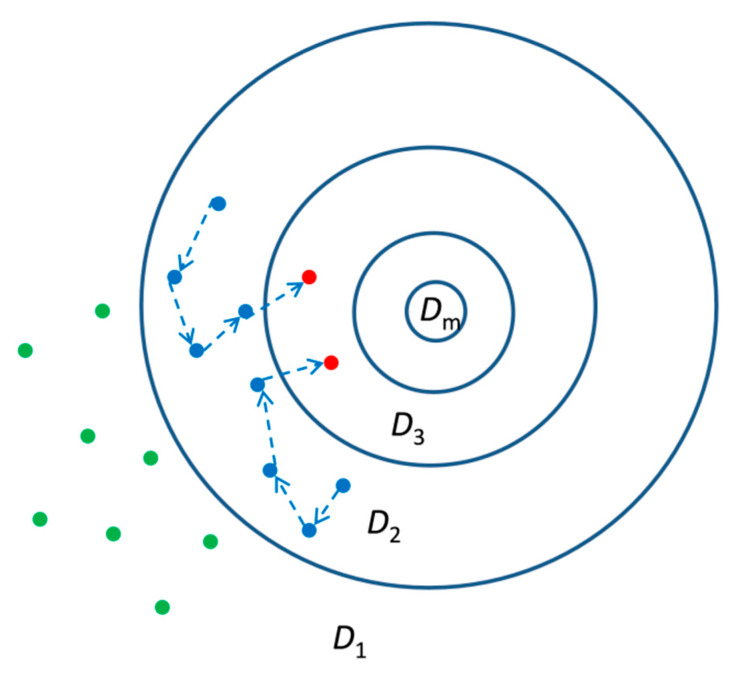
Schematic diagram of approximate Bayesian computation with subset simulation (ABC-SubSim).

To enhance the efficiency of ABC, a new algorithm called ABC-SubSim was conceived by Chiachio et al. [27]. In this algorithm, the subset simulation technique [28] is incorporated into the ABC framework, and the idea is to select the nested descending sequence in the subset simulation as the approximate region where the simulated dataset gets closer and closer to the observed data (Figure 1).

Let’s define *z* as z=(θ,x), so that p(z)=p(x|θ)p(θ). A sequence of nested decreasing regions *D_j_*, *j* = 1…, *m*, are defined as

(4) $$D_{j}=\left\{\left(\theta ,x\right):\rho \left(\eta \left(x\right),\eta \left(y\right)\right)\leq \epsilon_{j}\right\}$$

where $\epsilon_{j}$ is intermediate tolerance. The sequence of intermediate tolerances $\epsilon_{1},\epsilon_{2},\ldots ,\epsilon_{m}$, with $\epsilon_{j+1}<\epsilon_{j}$, are chosen adaptively which will be described later. The number of levels *m* is chosen so that $\epsilon_{m}\leq \epsilon$, a specified tolerance. Thus, it follows that

(5) $$p\left(D_{m}\right)=p\left(D_{1}\right)\overset{m}{\underset{j=2}{\prod }}p\left(D_{j}|D_{j-1}\right)$$

The samples of the first level are drawn using a direct Monte Carlo simulation from the prior distribution, while the samples of higher levels are drawn using a modified Metropolis algorithm (MMA) [28]. Instead of drawing a complete parameter vector candidate from a multidimensional proposal PDF as the original algorithm would do, MMA chooses a univariate proposal PDF for each component of the parameter vector, and the generated component candidate is accepted or rejected separately. It should be noted that although ABC-SubSim and ABC-SMC do have some similarities—they both have a series of intermediate tolerances; they differ in the way of generation of new samples from the samples in the previous level. In the ABC-SMC [25,33], samples are drawn from the previous population with some weights at first, and then they perturb the sample to obtain a new sample according to a perturbation kernel; while in the ABC-SubSim, the new samples are generated using the modified Metropolis algorithm from the seeds provided by the previous level.

#### 2.2.1. Intermediate Tolerances

In the actual implementation of subset simulation, the intermediate tolerances are adaptively chosen so that $p\left(D_{1}\right)$ and $p\left(D_{j}|D_{j-1}\right)$, *j* = 1, …, *m* are equal to a fixed value *P*_0_. In this way, the value of intermediate tolerance $\epsilon_{j}$ is determined as the average of the (*NP*_0_)th and (*NP*_0_ + 1)th values of the set of distances $\rho \left(\eta \left(x_{j-1}^{n}\right),\eta \left(y\right)\right)$, *n* = 1, …, *N*, arranged in increasing order, where *N* is the number of samples in each level. In this algorithm, the number of samples generated in *D_j_*_−1_ falling in *D_j_* is equal to *NP*_0_, and these *NP*_0_ samples are utilized as seeds to generate *NP*_0_ Markov chains with a length of 1/*P*_0_ samples, in which the new (1/*P*_0_ − 1) samples of each chain are produced by MMA [28]. For the sake of implementation, the numbers *NP*_0_ and 1/*P*_0_ are both integers. Thus, the total number of samples in *D_j_* is also *N*, of which *NP*_0_ samples are produced in the (*j*−1)th level.

#### 2.2.2. Conditional Probability

The parameter *P*_0_ determines the number of intermediate domains *D_j_* required to reach the target region *D*, which thereby influences the simulation efficiency. A smaller value of *P*_0_ connotes that fewer intermediate levels are required to reach *D*, but it makes for larger number of samples *N* required at each level to accurately estimate small conditional probabilities. On the other hand, a larger value of *P*_0_, although decreasing the number of samples required for each level, will increase the number of intermediate levels *m*. Therefore, we need to take a compromise value, and a theoretical study shows that the optimal choice is $P_{0}^{opt}\approx 0.2$ [34].

#### 2.2.3. Variance of Proposal Distribution

It was observed in [28] that the type of proposal PDFs (Gaussian, uniform, etc.) has no significant impact the efficiency of MMA, while their spread (variance) has a greater impact. In case of Gaussian proposal PDFs, too large or too small variances $\sigma_{j}^{2}$ tend to increase the correlation between successive samples. Larger variances may reduce the acceptance rate and increase the number of duplicate MCMC samples. Conversely, smaller variances may result in higher acceptance rates, but because they are closely related, the chain moves too slowly and produces many correlated samples. A theoretical study on the optimal variances for the proposal PDF has shown that the nearly optimal variances can be selected to make the acceptance rate between 30% and 50% at each conditional level [34]. Unfortunately, achieving this target acceptance rate usually requires the user’s prior experience or through inefficient brute force searches. Recently, Vakilzadeh et al. [29] proposed a self-regulating algorithm in which the proposal variance is adjusted adaptively in ABC-SubSim to realize effective sampling in the posterior PDF. By using this algorithm, the proposal variance can be dynamically adjusted to force the average acceptance rate to an ideal target value.

## 3. Model Updating Using ABC-SubSim

### 3.1. Choice of Metric Function

The quality of the posterior approximation depends on a suitable choice of the metric ρ and the tolerance parameter ϵ. In this subsection, the choice of the metric ρ is presented. In the following subsection, the tolerance parameter ϵ will be discussed. We choose the metric ρ for measuring the closeness of the model predicted modal data vector and the observed modal data vector as follows

(6) $$\rho \left(\eta \left(f_{r},\phi_{r}\right),\eta \left({\hat{f}}_{r}^{i},{\hat{\phi }}_{r}^{i}\right)\right)=\overset{N_{m}}{\underset{r=1}{\sum }}\left(w_{r}^{f}\overset{N_{s}}{\underset{i=1}{\sum }}{\left(\frac{f_{r}\left(\theta \right)-{\hat{f}}_{r}^{i}}{{\hat{f}}_{r}^{i}}\right)}^{2}+w_{r}^{\phi }\overset{N_{s}}{\underset{i=1}{\sum }}\frac{\|a_{r}^{i}\phi_{r}\left(\theta \right)-{\hat{\phi }}_{r}^{i}{\|}^{2}}{\|{\hat{\phi }}_{r}^{i}{\|}^{2}}\right)$$

where $w_{r}^{f}$ and $w_{r}^{\phi }$ are the weighting factors for the residues of the *r*th modal frequency and the *r*th mode shape, respectively; $f_{r}\left(\theta \right)$ is the *r*th model predicted modal frequency, ${\hat{f}}_{r}^{i}$ is the *r*th observed modal frequency in the *i*th data set; $\phi_{r}\left(\theta \right)$ is the *r*th model predicted partial mode shape confined on the observed degrees of freedom (DOFs), ${\hat{\phi }}_{r}^{i}$ is the *r*th observed mode shape in the *i*th data set, $a_{r}^{i}={\left({\hat{\phi }}_{r}^{i}\right)}^{T}\phi_{r}\left(\theta \right)/\left(\phi_{r}{\left(\theta \right)}^{T}\phi_{r}\left(\theta \right)\right)$ is a scaling factor that guaranties that the predicted model mode shape $\phi_{r}\left(\theta \right)$ is closest to the measured mode shape ${\hat{\phi }}_{r}^{i}$ at the measured DOFs, and *N_m_* and *N_s_* is the number of modes and number of observed data sets. The determination of the weighting factors $w_{r}^{f}$ and $w_{r}^{\phi }$ is discussed in details in Section 3.4. It should be noted that besides the metric presented above, one can also choose another function as the metric to measure the closeness of the mode prediction and the observations [9,35,36,37].

### 3.2. Stopping Criteria

The tolerance parameter ϵ is another important factor that controls the quality of the posterior approximation. In theory, when ϵ → 0, the algorithm can get an accurate posterior distribution, but this could not be achieved in practice. If one wants to get a good approximate posterior distribution in the ABC approach, ϵ has to be small enough so that the predicted outputs are accepted only if they fall into the nearest neighboring domain centered on the observation data vector. However, how small is sufficient is unknown in advance. By using a different metric, the tolerance level may be quite different to get a satisfactory accuracy. For example, if we use another set of weighting factors for the metric presented in Equation (6), for the same tolerance level ϵ, the accuracy of the posterior approximation may be quite different. In contrast, we set the stopping criteria by justifying the convergence of the model parameters through looking at two relative indexes. The new stopping criteria to cease the subset simulation are as follows:

(7) $$R_{j}=\frac{\rho_{j}^{max}-\rho_{j}^{min}}{\rho_{j}^{min}}\leq R^{tol}$$

(8) $$S_{j}=\frac{\left\|\theta_{j}^{opt}-\theta_{j-1}^{opt}\right\|}{\left\|\theta_{2}^{opt}-\theta_{1}^{opt}\right\|}\leq S^{tol}$$

where $\rho_{j}^{max}$ and $\rho_{j}^{min}$ are the maximum and minimum values of the metric in the *j*th conditional level, respectively; $R^{tol}$ is the tolerance of *R*, which can be chosen as a very small value (e.g., 10^−3^); $\theta_{j}^{opt}$ and $\theta_{j-1}^{opt}$ are the optimal model parameter vector in the *j*th and (*j* − 1)th conditional levels, respectively; and $S^{tol}$ is the tolerance of *S*, which can be chosen as a relatively larger value (e.g., 10^−2^). If both the stopping criteria in Equations (7) and (8) are satisfied, the subset simulation is stopped. *R_j_* is defined to measure the diversity of the *j*th conditional level, and *S*_j_ is defined as the improvement of the *j*th conditional level. In such a way, although we have to specify two tolerance values, the meanings behind these two values are more clear and straightforward, and this approach is more operable in practice. It should be noted that this is only an expedient. To completely solve this problem, we still need to deeply study the relationship between likelihood-informed Bayesian inference and likelihood-free approximate Bayesian computation so as to provide a theoretical basis for selecting the appropriate tolerance [38].

### 3.3. Hybrid Optimization Scheme

Stochastic sampling techniques such as subset simulation have the advantage of efficient posterior sampling capacity, but their ability of finding an optimum or “maximum a-posteriori” is relatively low. The stochastic sampling techniques can reach the region near an optimum point relatively quickly, but it can take many levels (generations) to achieve the convergence to the exact optimum. Therefore, in order to improve the overall search ability, it is necessary to combine a powerful global search sampler with an efficient local search operator to develop a hybrid scheme. For example, Jung and Kim proposed a hybrid genetic algorithm to update their structural model by combining the genetic algorithm and the improved Nelder–Mead’s simplex method [39]. Sun and Betti proposed a hybrid optimization algorithm that combines a modified artificial bee colony (MABC) algorithm and the Broyden-Fletcher-Goldfarb-Shanno (BFGS) method for Bayesian model updating [40]. As a global stochastic sampler, the subset simulation is able to quickly reach the approximation region of the posterior for a given tolerance level, and this region stands for the approximate posterior distribution of the model parameter vector *θ*. However, the optimal value given by the subset simulation may not be the “exact” optimum, and this “exact” optimum can be rapidly refined by a local optimization search operator.

In this study, the BFGS method was used to fine-tune the optimal solution given by the subset simulation to obtain a better solution. Herein, in order to make sure that the hybrid scheme did not disturb the stochastic process of the subset simulation, the BFGS method was just introduced at the final level of the subset simulation. The optimal value at the final level of the subset simulation was taken as the initial guess in the BFGS method. The BFGS method is a gradient-based quasi-Newton method that uses the BFGS approximation of the hessian matrix. The details of the BFGS method can be found in the literature [41,42,43,44]

### 3.4. Weighting Factors Determination

In the metric function as presented in Equation (6), weight factors are imposed a relative difference between the modal frequency and the mode shape, as well as between different modes, because the measurement precision of these quantities may be different. The identification accuracy of experimental modal frequencies is usually higher than that of modal shapes, so appropriate weights are necessary. In the literature, most of them chose these weighting factors by engineering experience [9,45]. A rigorous theoretical study on the optimal weighting factors in the weighted modal residuals metric for structural model updating was conducted by Christodoulou and Papadimitriou [46]. The results showed that the optimal weight of the modal residual is asymptotically inversely proportional to the value of the modal residual at the optimal model parameters when there is a large amount of measured data. The weighted modal residuals metric in Equation (6) can be rewritten as:

(9) $$J\left(\theta ,w\right)=\overset{n}{\underset{i=1}{\sum }}w_{i}J_{i}\left(\theta \right)$$

where $J_{i}\left(\theta \right)$ is the residual of a specific modal group and $w_{i}$ is its weighting factor. The optimal weights in Equation (6) are given by

(10) $${\hat{w}}_{i}=\frac{\alpha_{i}}{J_{i}\left({\hat{\theta }}_{opt}\right)}$$

where $\alpha_{i}$ is a scale reflecting the proportion of the data volume contained in each modal group to the total data volume in a modal data set. *N_m_* denotes the number of modes, and *N_o_* denotes the number of observed degree of freedom; as such, the total number $N_{t}=\left(N_{o}+1\right)N_{m}$ and $\alpha_{i}=1/N_{t}$ for modal frequency, while $\alpha_{i}=N_{o}/N_{t}$ for mode shape, satisfying $\sum_{i=1}^{n}\alpha_{i}=1$. However, the optimal weighting factors given in Equation (10) is dependent on the optimal model parameter vector ${\hat{\theta }}_{opt}$, which is unknown in advance. Therefore, one should solve it in an iterative approach. The initial value for the weighting factors can be calculated using Equation (10) when taking the nominal value of the model parameter vector as its optimal value ${\hat{\theta }}_{opt}$. By using ABC-SubSim and the BFGS method, a new optimal value for ${\hat{\theta }}_{opt}$ is obtained, and then the new weighting factors can be calculated using Equation (10) again. These procedures are repeated several times until the convergence satisfied.

### 3.5. Summary of the Procedures

The proposed algorithm for probabilistic model updating can be schematically described below:

- Calculate the initial values for the weighting factors used in the metric as presented in Equation (6) using Equation (10) when taking the nominal value of the model parameter vector as its optimal value.
- Using direct Monte Carlo simulation to get *N* samples following the prior distribution of the model parameter vector.
- Evaluate the metric and sort the samples in order according to their metric values.
- Select *NP*_0_ samples which corresponding to the first *NP*_0_ smallest metric values. These samples will be taken as the seeds for Markov chains generation.
- Generating *NP*_0_ Markov chains with length of 1/*P*_0_ for each chain using a self-regulating modified Metropolis algorithm [29].
- Check whether the stopping criteria proposed in Equations (7) and (8) is satisfied. If it is not satisfied then repeat the steps iii–vi. If it is satisfied then output the posterior samples.
- Take the optimal value in the posterior samples as the initial guess for a local optimization search operator, and conduct the local optimization search to obtain a fine optimum.
- Using the fine optimum to calculate the weighting factors using Equation (10).
- Check the convergence of the weighting factors. If the convergence satisfied, then the algorithm ends. If not, take the newly calculated weighting factors for the metric and repeat the steps i–ix.

**Figure 2:**
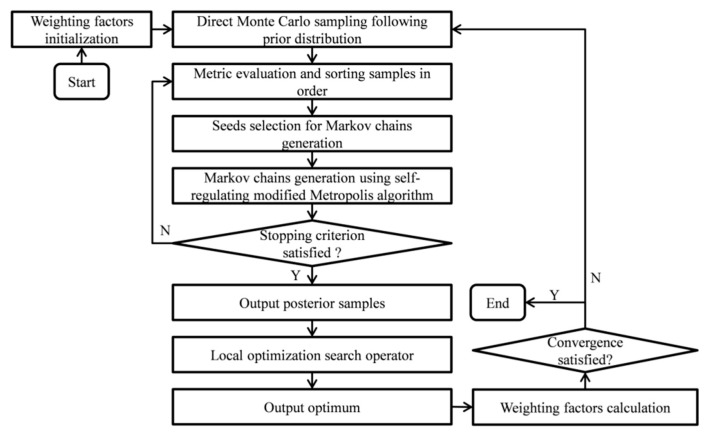
The flowchart of the proposed algorithm.

A flowchart of the proposed algorithm is given in Figure 2, and a pseudocode is provided in Appendix A.

## 4. Damage Assessment Based on Model Updating

### 4.1. Structural Model Parameterization

For model updating and damage assessment of linear structures, the structure is usually partitioned into $N_{\theta }$ substructures, and a substructure can be a group of elements or just one single element. The global stiffness matrix is parameterized as follows:

(11) $$K\left(\theta \right)=K_{0}+\overset{N_{\theta }}{\underset{n=1}{\sum }}\theta_{n}K_{n}$$

where *K*_0_ is the global stiffness matrix before updating, *K*_n_ is the nominal stiffness matrix of the *n*th substructure, and *θ*_n_ is a perturbation scaling factor used to change the nominal substructure stiffness *K*_n_ so that the updated model is more in line with the actual behavior of structure. When a substructure is damaged, the optimal value of the parameter *θ*_n_ decreases. The mass matrix can be parameterized in a similar substructural manner, but mass updates were not considered here, because the main interest of this paper was damage assessment, and the mass was assumed to be known accurately enough and to not change with the occurrence of damage.

### 4.2. Probabilistic Damage Assessment

According to a two-stage procedure for structural damage assessment using Bayesian model updating proposed by Ching and Beck [47], experimental modal parameters are identified from measured data at intact and damaged state in the first stage; in the second stage, the PDFs of structural stiffness parameters are updated using the Bayesian approach before and after damage, and the updated PDFs are used to evaluate the probability of damage of each substructure. Assuming that the stiffness parameters in the intact state and the possible damaged state are conditionally independent (and by using Gaussian asymptotic approximation), the probability of stiffness reduction of parameter *θ*_n_ which has been reduced by certain fraction *d* compared with the undamaged state can be obtained:

(12) $$P_{n}^{dm}\left(d\right)=P[\left(1+\theta_{n}^{pd}\right)<\left(1-d\right)\left(1+\theta_{n}^{ud}\right)]\approx \Phi \left(\frac{\left(1-d\right)\left(1+{\hat{\theta }}_{n}^{ud}\right)-\left(1+{\hat{\theta }}_{n}^{pd}\right)}{\sqrt{{\left(1-d\right)}^{2}{\left({\hat{\sigma }}_{n}^{ud}\right)}^{2}+{\left({\hat{\sigma }}_{n}^{pd}\right)}^{2}}}\right)$$

where Φ( ) is the cumulative distribution function of the standard normal distribution; ${\hat{\theta }}_{n}^{ud}$ and ${\hat{\theta }}_{n}^{pd}$ stand for the a maximum a posterior probability (MAP) estimate of the stiffness parameters at the undamaged state and possibly damaged state, respectively; and ${\hat{\sigma }}_{n}^{ud}$ and ${\hat{\sigma }}_{n}^{pd}$ denote the corresponding standard deviations of the stiffness parameters.

## 5. Application Examples

### 5.1. Model Updating of a Shear Building

An eight-story shear building was considered in this example. The mass per floor was assumed to be 2 × 10^4^ kg, while the nominal inter-story stiffness for all floors was assumed to be 15 × 10^6^ N/m. The actual value of the inter-story stiffness for the first four stories had a 20% reduction of the nominal ones, while that for the remaining four stories had a 20% increase of the nominal ones. We denoted the model parameters vector *θ* as the increment ratio of the inter-story stiffness, compared to their nominal ones, so their true values were *θ* = {−0.2, −0.2, −0.2, −0.2, 0.2, 0.2, 0.2, 0.2}. Twenty sets of modal data were assumed to be available for the structural model updating, where the incomplete measurements were considered; that is, only five floors (1, 3, 5, 7, and 8) were measured, and only the first four modes were identified. For the simulated modal data, zero-mean Gaussian noise with a diagonal covariance matrix $\Sigma_{\varepsilon }$ was added to the exact calculated modal frequencies and mode shapes. Two cases of noise level were considered in this study. In noise level 1, a 1% coefficient of variation (CV) noise was added to the modal frequencies, while a 5% of CV noise was added to the mode shapes for all modes, a reasonable value for typical modal tests. Noise level 2 was a more severe noise level situation, where 2% of CV noise was added to the modal frequencies, while 10% of CV noise was added to the mode shapes for all modes. In order to study the effect of the BFGS method on the accuracy of the results in the fine-tuning stage, the results before and after the fine-tuning were compared. Moreover, two sets of different values for the stopping criteria parameters were used to study their impact on the final results. In tolerance level 1, we set the stopping criteria parameters *R*_tol_ = 10^−3^ and *S*_tol_ = 10^−2^, while in tolerance level 2, stricter stopping criteria parameters were used: *R*_tol_ = 10^−4^ and *S*_tol_ = 10^−3^. Totally, there were four cases in the simulations: case 1 with noise level 1, fine-tuning, and tolerance level 1; case 2 with noise level 2, fine-tuning, and tolerance level 1; case 3 with noise level 1, without fine-tuning, and tolerance level 1; and case 4 with noise level 1, fine-tuning, and tolerance level 2.

We used the method proposed in Section 3 to update the stiffness parameters vector *θ*. In the subset simulation, we chose the number of samples in each level *N* = 2000 and the level conditional probability *P*_0_ = 0.2. The variance in Gaussian proposal distribution was regulated using a self-regulating algorithm proposed by Vakilzadeh et al. [29].

**Figure 3:**
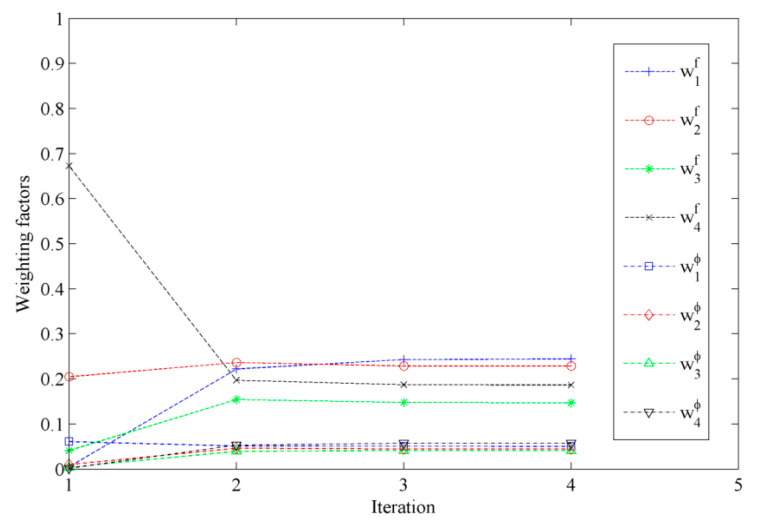
The evolution of the optimal weighting factors with iteration steps (case 1).

The optimal weighting factors in the metric are obtained using an iterative approach. The evolution of the optimal weighting factors with the iteration steps is shown in Figure 3. It can be seen that the weighting factors converge very fast. It also can be seen that the weighting factors for the modal frequency are larger than those for the mode shape, which means that more uncertainness are contained in the mode shape. It agrees with our preset simulated modal data, in which we added lager noise to the mode shapes than the modal frequencies.

**Figure 4:**
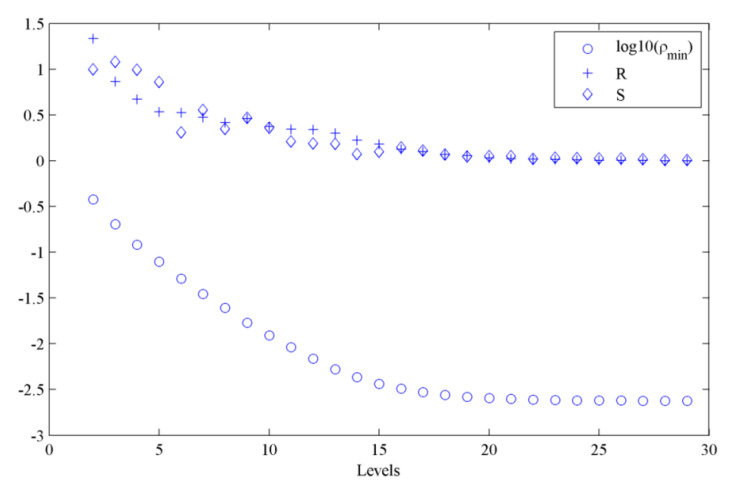
The convergence of the metric and the model parameters vector with the subset levels (case 1).

The convergence of the metric and the model parameters vector with the subset levels when using the optimal weighting factors in the metric for case 1 is shown in Figure 4, where *R* and *S* are the quantities defined in Equations (7) and (8) to measure the convergence of the metric and the model parameters vector, respectively, and log10 (*ρ*_min_) is the logarithm of the minimum value of the metric in each level. It can be seen that the convergence parameters (*R*, *S*) nearly became flat from the 20th level, and the subset simulation stopped at the 29th level based on our preset tolerances of *R*_tol_ and *S*_tol_.

**Figure 5:**
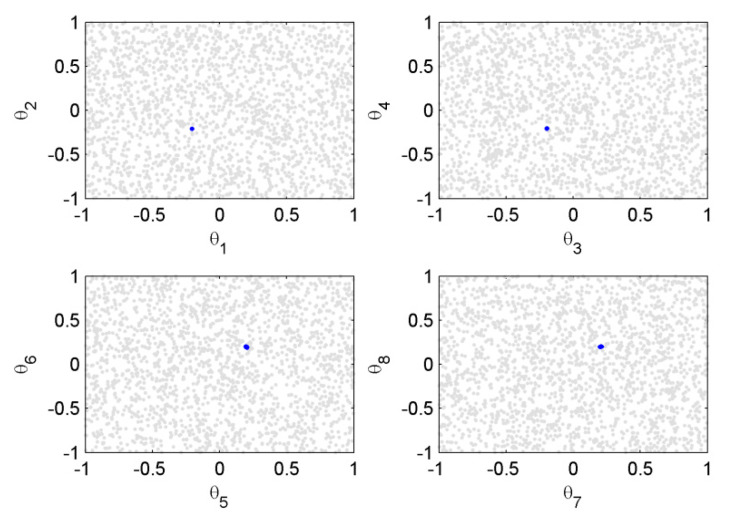
Scatter plot graph of posterior (in blue) and prior (in gray) samples of the model parameters (case 1).

**Figure 6:**
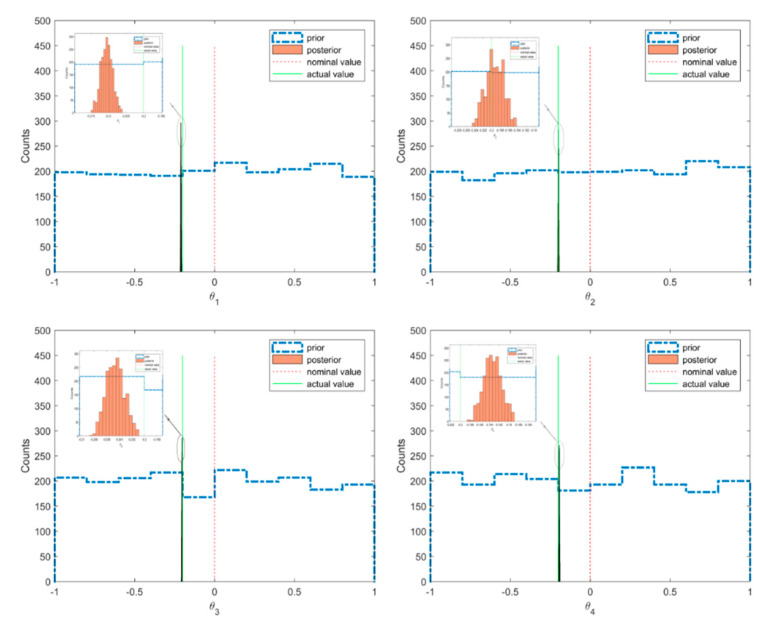

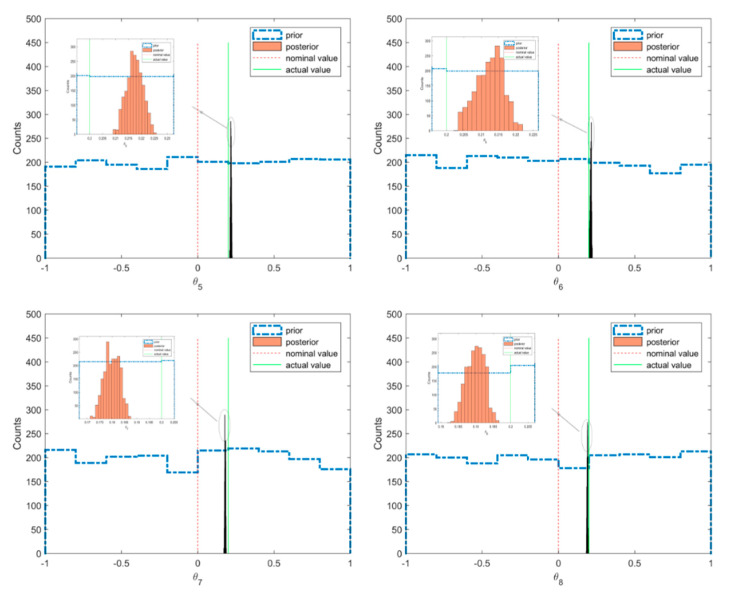
Prior and posterior samples of the model parameters with actual and nominal values (case 1).

The scatter plot of the posterior and prior samples for case 1 are shown in Figure 5, where the prior distributions of the model parameters were taken as uniform distribution within the interval [−1,1]. It can be seen that the posterior samples were confined in very small regions compared with the prior samples. This means that when using Bayesian inference from the informative measured modal data, the uncertainties of the model parameters contained in the posterior distribution were reduced much more compared to the prior distribution. The histograms of the prior and posterior samples of the model parameters are provided in Figure 6. It can be seen that the posterior samples were concentrated in a smaller range compared to the prior samples, leading to a peaked posterior PDF around the actual values of −0.2 or 0.2.

The updated results of the model parameters are tabulated in Table 1. It can be seen that the optimal values identified in case 1 were very close to their true values. Though the accuracy of the results in case 2 was not as good as that in case 1, it was also acceptable. On the other hand, it can be seen that the coefficients of variation (CV) of the model parameters identified in case 2 were larger than those in case 1, which means that more uncertainties were involved in the identified model parameters based on the modal data in case 2. Comparing case 3 and case 1, it could be seen that the accuracy of the optimal values was improved through fine-tuning with the BFGS method. The coefficients of variation were reduced in case 4, which means that the uncertainty could be reduced by setting a stricter tolerance level for the stopping criteria.

### 5.2. Damage Assessment of a Truss Structure

**Figure 7:**
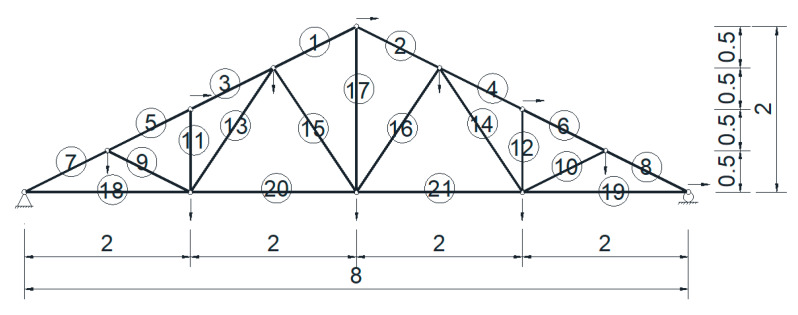
Schematic of a 21-bar planar truss (unit: m).

A 21-bar planar truss model is shown in Figure 7, and the structure was simply supported with a span of 8 m. The cross sectional area of each bar was 100 mm^2^. The mass density was 7850 kg/m^3^. The actual value of the elastic modulus was 190 GPa, and the nominal value was set to be 200 GPa. This means that a stiffness reduction factor of 5% was assumed for each bar element in the undamaged state, while additional stiffness reductions of 20% and 40% were assumed for bar 20 and 21, respectively. This means that the damage extents of bar #20 and #21 at the damage state were 21% (0.2/0.95) and 42% (0.4/0.95), respectively. The structure had 12 nodes, 21 elements, and 21 DOFs. It was assumed that only the first eight modes were available, and only 11 DOFs were measured (marked with arrows in Figure 7). Some nodes were only measured in the vertical direction, and some nodes were only measured in the horizontal direction. This means that the measurements were incomplete, both in modes and DOFs, which was also the case in real situation. It was also assumed that there were 20 data sets of modal frequencies and mode shapes contaminated with white noise of 1% and 5%, respectively.

**Figure 8:**
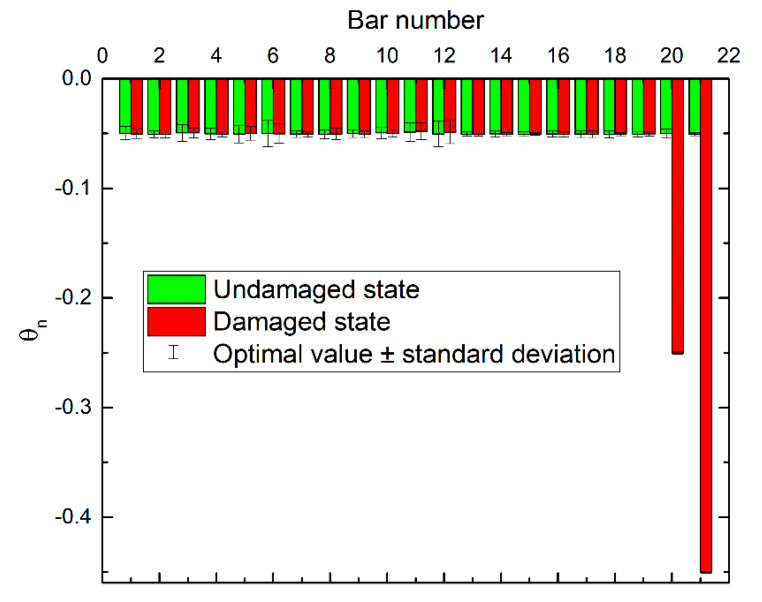
Updated perturbation scaling factors of stiffness for truss model.

Using the model updating method proposed in Section 3, the updated results of the perturbation scaling factors of stiffness *θ* are shown in Figure 8, both in the undamaged and damaged states. It can be seen that the optimal values of the updated parameters were very close to their true values. Their standard deviations were also obtained by the posterior samples that corresponded to the samples in the last level of subset simulation. From the figure, it can be seen that bars #6 and #12 had relatively larger standard deviations than the others, which means that more uncertainties were involved in these two bars based on the current sensor configuration. This reflects the fact that the information contained in the monitoring data, which was critical for damage detection, was dependent on the sensor layout. This was a sensor placement optimization issue, which is not the subject of this article, so readers can refer to some literature on this subject [48,49].

**Figure 9:**
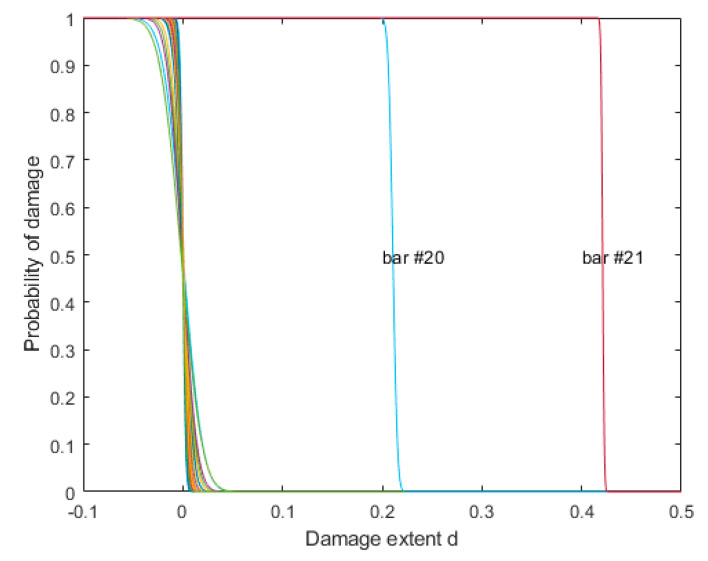
Probability curve of damage extent for truss model.

Using the damage assessment method proposed in Section 4, the probability curve of damage extent for each bar is shown in Figure 9. This figure clearly shows the probability of damage extent defined in Equation (12). It can be seen that there were damages with extents of 21% and 42% in bar #20 and #21, respectively, which agreed well with our preset values.

### 5.3. Damage Assessment of a Simply Supported Beam

**Figure 10:**
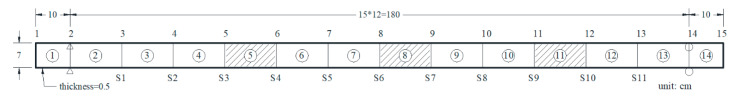
Simply supported beam model.

A simply supported steel beam before and after damages at different locations was used for further verification. The beam and the sensor layout (marked with S1~S11) are shown in Figure 10. The total length of the beam was 200 cm, and the span length was 180 cm. The width and the thickness of the beam were 7 and 0.5 cm, respectively. For the finite element modeling of the beam, 15 nodes and 14 elements were used, where two ends, two supports, and eleven sensor locations were set as nodes. Only vertical displacements at the sensor locations were measured, while the rotations were difficult to be measured in the real case. The mass per unit volume was 7850 kg/m^3^. The true value of the modulus of elasticity was 190 GPa, while the nominal value was set to be 200 GPa, which means a stiffness reduction factor of 5% was assumed for each element in the undamaged state. It was also assumed that 20 data sets of modal frequencies and mode shapes of the first five modes contaminated with white noise of 1% and 5%, respectively, were available. The damages were introduced in elements 5, 8, and 11, where the extents were 10%, 20%, and 30% (compared with undamaged state not nominal model), respectively.

**Figure 11:**
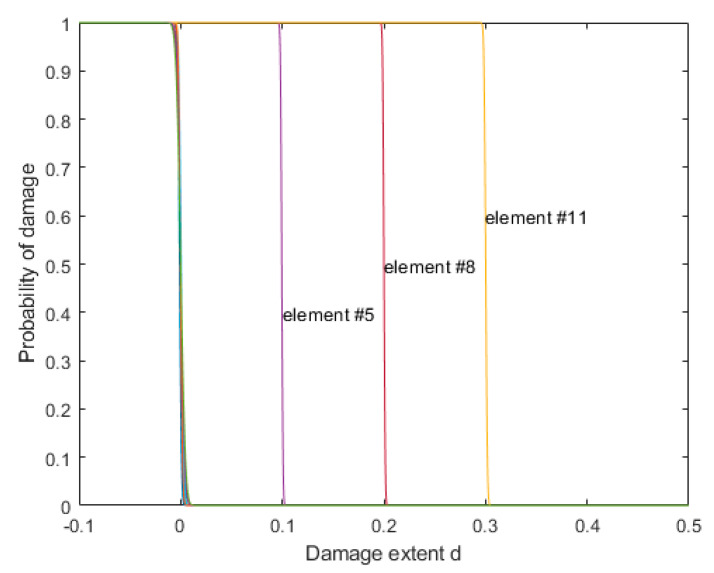
Probability curve of damage extent for beam model.

By using the proposed model updating algorithm in Section 3 and the damage assessment method proposed in Section 4, the probability curve of damage extent for each element is shown in Figure 11. It can be seen that there were damages with extents of 10%, 20%, and 30% in elements 5, 8, and 11, respectively, which agreed well with the predefined damaged model.

## 6. Conclusions

A structural model updating and damage assessment method using the ABC-SubSim algorithm was presented. An iterative procedure to find the optimal weighting factors related to different modal residuals and a new stopping criterion to find a proper tolerance level were proposed for the metric used in the ABC-SubSim. A hybrid optimization scheme was proposed to identify the structural parameters based on incomplete modal data with uncertainty. A high accuracy of the structural model updating is guaranteed by the proposed ABC-SubSim algorithm, even though the noise level of modal data was up to 10%. Three illustrative examples well-demonstrated the effectiveness and reliability of the proposed algorithm. Possible directions of future research related to the ABC method may be a relation study between likelihood-informed Bayesian inference and likelihood-free approximate Bayesian computation, thus leading to a more reasonable selection of key parameters in the ABC method.

## Figures and Tables

**Table 1:** The identified results of the model parameters.

	*θ*_1_	*θ*_2_	*θ*_3_	*θ*_4_	*θ*_5_	*θ*_6_	*θ*_7_	*θ*_8_
True	−0.2000	−0.2000	−0.2000	−0.2000	0.2000	0.2000	0.2000	0.2000
Optimal ^1^	−0.2052	−0.2085	−0.1968	−0.2050	0.2002	0.1968	0.2052	0.2000
CV ^1^	0.0059	0.0072	0.0063	0.0084	0.0126	0.0165	0.0173	0.0063
Optimal ^2^	−0.1930	−0.2058	−0.2067	−0.2044	0.2184	0.2144	0.2104	0.2011
CV ^2^	0.0135	0.0157	0.0120	0.0165	0.0240	0.0336	0.0361	0.0120
Optimal ^3^	−0.2121	−0.2007	−0.2050	−0.1920	0.2167	0.2159	0.1799	0.1848
CV ^3^	0.0059	0.0072	0.0063	0.0084	0.0126	0.0165	0.0173	0.0063
Optimal ^4^	−0.1992	−0.1969	−0.2005	−0.2102	0.1941	0.1863	0.2043	0.2029
CV ^4^	0.0005	0.0008	0.0004	0.0005	0.0005	0.0015	0.0011	0.0010

Note: Optimal *^i^* and CV *^i^* denote the optimal values and the coefficients of variation of the model parameters identified in case *i* (*i* = 1,2,3,4).

## Appendix A

A pseudocode for the proposed algorithm as described in Section 3.5 is provided below.

**The proposed ABC-SubSim algorithm for structural model updating using modal data**
Input
*M*, *K*_0_: the mass and nominal stiffness matrix
*y* = {${\hat{f}}_{r}^{i}$, ${\hat{\phi }}_{r}^{i}$}: the measured frequencies and mode shapes, *i* = 1:N_s_, *r* = 1:N_m_
*θ*_0_, *p*(*θ*): the nominal value and prior PDF of model parameters
Output
*θ_opt_*, Θ*_post_*: the optimal value and posterior samples of model parameters
/*Initialization*/
$R^{tol}$, $S^{tol}$: tolerances for stopping criterion of subset simulation
*P*_0_, *N*: the conditional probability and the number of samples in each level of subset simulation
*w*_0_: calculate the initial values for the weighting factors based on nominal value *θ*_0_
*ε**_w_*: tolerances for stopping criterion of weighting factors iteration
*k* = 1
**while** (*k*==1 or $\left|w_{k}-w_{k-1}\right|/w_{k-1}>\varepsilon_{w}$) **do /***External loop for weighting factors iteration*/
Sample $\left[\left(\theta_{0}^{\left(1\right)},x_{0}^{\left(1\right)}),\ldots ,(\theta_{0}^{\left(n\right)},x_{0}^{\left(n\right)}),\ldots ,(\theta_{0}^{\left(N\right)},x_{0}^{\left(N\right)}\right)\right]$, where (θ,x)~p(θ)p(x|θ)
*j* = 1
**while** (*j*==1 or *R_j_* >$R^{tol}$ and *S_j_* > $S^{tol}$) **do /***Internal loop for subset simulation*/
**for** *n*: 1, …, *N* **do**
Evaluate $\rho_{j}^{\left(n\right)}=\rho \left(\eta \left(x_{j-1}^{\left(n\right)}\right),\eta \left(y\right)\right)$
**end for**
Sort and renumber the samples $\left[(\theta_{j-1}^{\left(n\right)},x_{j-1}^{\left(n\right)}),n:1,\ldots ,N\right]$ so that $\rho_{j}^{\left(1\right)}\leq \rho_{j}^{\left(2\right)}\leq \cdots \rho_{j}^{\left(N\right)}$
$$\epsilon_{j}=\frac{1}{2}\left(\rho_{j}^{\left(NP_{0}\right)}+\rho_{j}^{\left(NP_{0}+1\right)}\right)$$
**for** *l* = 1, …, $NP_{0}$ **do**
Select as a seed $(\theta_{j-1}^{\left(l\right)},x_{j-1}^{\left(l\right)})\in D_{j}$
Run a self-regulating modified Metropolis algorithm [29] to generate 1/$P_{0}$ states of a Markov chain lying in $D_{j}$: $\left[\left(\theta_{j}^{\left(l\right),1},x_{j}^{\left(l\right),1}),\ldots ,(\theta_{j}^{\left(l\right),1/P_{0}},x_{j}^{\left(l\right),1/P_{0}}\right)\right]$
**end for**
Renumber $\left[(\theta_{j}^{\left(l\right),m},x_{j}^{\left(l\right),m}),l=1,\ldots ,NP_{0};m=1,\ldots ,1/P_{0}\right]$ as $\left[\left(\theta_{j}^{\left(1\right)},x_{j}^{\left(1\right)}),\ldots ,(\theta_{j}^{\left(N\right)},x_{j}^{\left(N\right)}\right)\right]$
*j* = *j* + 1
Update *R_j_ and S_j_*
**end while**
*k* = *k* + 1
Take the optimal value in the last level of subset simulation samples as the initial guess for a local optimization search operator, and conduct the local optimization search to obtain a fine optimum.
Update the weighting factors $w_{k}$ based on the newly obtained optimal model parameters
**end while**
